# Sleep and Circadian Rhythms in Patients with Physical and Mental Disorders

**DOI:** 10.3390/jcm15124774

**Published:** 2026-06-19

**Authors:** Argyro Pachi, Ioannis Ilias, Athanasios Tselebis

**Affiliations:** 1 Psychiatric Department, Sotiria Thoracic Diseases Hospital of Athens, 11527 Athens, Greece; irapah67@gmail.com; 2 Department of Endocrinology, Hippokration General Hospital of Athens, 11527 Athens, Greece; iiliasmd@yahoo.com

Sleep is not a passive state of rest but a biologically active process essential for cognitive function, immune regulation, emotional homeostasis, and metabolic integrity. Disruptions to sleep or the circadian rhythms that orchestrate it are now understood to be both consequences and drivers of a broad spectrum of physical and mental health conditions [1,2]. Their relationship is bidirectional and often self-reinforcing: illness fragments and shortens sleep; in turn, disordered sleep worsens the course and prognosis of the underlying condition. Over the past decade, converging evidence from chronobiology, behavioral medicine, and clinical psychiatry has made it increasingly clear that addressing sleep and circadian dysregulation is not peripheral to patient care but central to it.

This Special Issue of the *Journal of Clinical Medicine* was conceived in order to consolidate current knowledge at the intersection of sleep, circadian, and behavioral medicine, chronotherapy, consultation–liaison psychiatry, and cognitive behavioral therapy for insomnia (CBT-I). Over its publication cycle, eight original articles and reviews were published, spanning populations as diverse as healthcare workers and individuals with Long COVID, narcolepsy, and bruxism or disordered eating. The eight contributions collectively map a field that has matured considerably yet retains numerous unresolved questions, the answers to which will shape clinical practice for years to come.

Despite the well-established epidemiology of insomnia affecting 10–30% of the general population and carrying elevated risks for depression, anxiety, substance use, cognitive impairment, and cardiovascular disease [3,4], several domains remained inadequately studied when this Special Issue was launched. First, the neuropsychiatric sequelae of Long COVID, an emerging condition affecting approximately 10% of those infected with SARS-CoV-2 [5], had received insufficient attention with respect to insomnia specifically. Second, while the occupational health of healthcare workers had been studied during the pandemic, the interplay of trait psychological variables such as anger, cynicism, and nightmare distress with insomnia required systematic exploration [6]. Third, non-pharmacological interventions for insomnia, particularly digital CBT-I and novel delivery contexts, had not been adequately examined in terms of contextual moderators and real-world feasibility [7]. Fourth, the intersection of circadian phenotype (chronotype) with psychiatric vulnerability, eating disorder risk, and thermoregulatory physiology had not been synthesized within a single clinical framework. Finally, behavioral interventions for narcolepsy, despite guideline endorsement, lacked an adequate evidence base.

Undergirding all of these gaps was a broader theoretical deficit: the absence of an integrated, translational model connecting circadian disruption to mental health vulnerability. Walker et al. [8] had demonstrated through a comprehensive review that disrupted circadian rhythms, whether induced by shift work or jetlag (social or travel-based), strongly correlate with affective and psychiatric disorders, operating via shared substrates in the limbic system, monoaminergic neurotransmission, and the hypothalamic–pituitary–adrenal axis. Also, recent research on the neurophysiological mechanisms underlying sleep disturbances across psychiatric disorders advances a transdiagnostic framework for understanding how changes in sleep architecture and circadian regulation interact with the neural circuits involved in major psychiatric disorders [9]. Specific electroencephalographic markers relevant to mood, anxiety, psychotic, and neurodevelopmental disorders point to disorder-specific sleep profiles. Moreover, findings from neuroimaging and genomics provide support for the convergence of sleep-related mechanisms with emotional and cognitive dysfunction. This Special Issue sought to populate the clinical branches of this framework with further data and perspectives.

In a longitudinal, multidimensional study of insomnia in patients with Long COVID [10], a large proportion of these patients met the criteria for clinically significant insomnia, which persisted during the nine-month follow-up regardless of the initial severity of COVID-19, suggesting that post-infectious insomnia is driven by neuropsychiatric and psychosocial mechanisms rather than by the acute somatic burden of illness. Insomnia severity correlated with anxiety, depression, perceived stress, and reduced cognitive function, a pattern consistent with a transdiagnostic process rather than a disease-specific sequela. The finding that spontaneous resolution is clinically insufficient, and that the polysomnographic profile resembles chronic primary insomnia, provides a direct rationale for proactive CBT-I trials in this population.

Two articles from Greece jointly addressed the mental health and sleep landscape of hospital nurses in the post-pandemic period [11,12], both employing mediation modeling to reveal that insomnia among healthcare workers is embedded in a complex psychosocial architecture rather than being a simple consequence of workload. More specifically, one study identified a pathway from occupational stress through depressive symptoms and burnout to insomnia, while the other implicated anger, cynical distrust, and nightmare distress as additional sequential mediators—with anger emerging as the strongest individual predictor of insomnia. These findings argue that routine sleep hygiene advice is inadequate for this population, and that effective intervention must target the upstream psychological and relational determinants of insomnia in occupational settings.

In a multicenter randomized controlled trial, digital CBT-I (dCBT-I) delivered within a structured crenotherapy context was evaluated versus stand-alone dCBT-I [13], with both groups achieved significant reductions in insomnia severity, consistent with the established efficacy of dCBT-I. While the primary between-group comparison did not demonstrate a statistically superior effect of the crenotherapy context on overall insomnia scores, exploratory analyses indicated that participants under 60 years old and those with comorbid anxiety symptoms derived greater benefit from the embedded delivery model. The study makes a methodological contribution by introducing the concept of contextual embedding, integrating digital interventions within structured medical environments to facilitate implementation, adherence, and psychosocial synergy, as a candidate paradigm for optimizing insomnia treatment in real-world settings.

A comprehensive review of thermoregulation across six sleep disorders—ADHD, insomnia, narcolepsy, obstructive sleep apnea, depression, and restless legs syndrome [14]—synthesized evidence around three parameters, namely, core body temperature (CBT), distal–proximal skin temperature gradient (DPG), and melatonin timing, to delineate disorder-specific thermoregulatory profiles and their therapeutic implications. Both ADHD and insomnia are characterized by delayed CBT minima and impaired distal heat dissipation, narcolepsy by an elevated DPG at all hours, depression by blunted circadian temperature amplitude, and OSA primarily by fragmented temperature rhythms secondary to intermittent hypoxia. The review proposes a disorder-specific framework for thermal interventions, noting that localized distal warming reduces sleep-onset latency in insomnia and ADHD, while whole-body hyperthermia is more appropriate for depression, and proximal warming may counteract excessive sleepiness in narcolepsy. This translational synthesis opens a concrete avenue for non-pharmacological chronotherapy.

A scoping review of psychological and behavioral interventions for narcolepsy was conducted, with only six studies meeting the inclusion criteria, despite guideline endorsement of non-pharmacological strategies, reflecting a profound evidence gap [15]. The included interventions—scheduled napping, CBT adapted for hypersomnolence (CBT-H), and mindfulness-based approaches—showed promising signals, particularly for reducing daytime sleepiness and depressive symptoms. Extended mindfulness programs (12 weeks) achieved clinically meaningful improvements across anxiety, depression, social functioning, and hypersomnia-related impairment. The authors call for larger randomized trials, standardized outcome measures, longer follow-up periods, and specific attention to pediatric and adolescent populations, in whom narcolepsy frequently debuts.

The associations between bruxism, insomnia, parasomnias, and dream content in adults were examined in a cross-sectional study [16]. A significant dose–response pattern was found, with greater bruxism severity associated with higher insomnia scores, more frequent parasomnias, and more frequent dreams involving oral sensations. The findings place bruxism not as an isolated stomatological concern but as part of a broader spectrum of sleep- and stress-related phenomena, suggesting that multidisciplinary clinical evaluation and treatment may benefit patients exhibiting any one component. Bruxism’s bidirectional relationship with insomnia, in which one condition may exacerbate the other through shared mechanisms involving nocturnal arousal and emotional dysregulation, was highlighted as a priority for future research.

Finally, the intersection of chronotype, body image, and eating disorder (ED) risk in adults was investigated in a cross-sectional study [17]. Evening chronotype was significantly associated with higher ED risk and more negative body image, and moderation analysis revealed that body image amplified the chronotype–ED risk relationship: among those with a strongly negative body image, eveningness was a powerful predictor of disordered eating, whereas among those with a positive body image the association was attenuated. This study extends the circadian medicine framework into the domain of eating disorders, reinforcing the broader thesis that circadian misalignment is a transdiagnostic vulnerability factor with implications across psychiatric and somatic conditions.

The eight articles in this Special Issue make meaningful contributions across several domains. The clinical profile of Long COVID insomnia was characterized in detail, providing evidence that this is a persistent, functionally significant condition that warrants proactive intervention rather than watchful waiting. Occupational insomnia in healthcare workers was shown to be not simply a product of workload but of a psychosocial cascade involving anger, cynicism, nightmare distress, burnout, and depression—a finding with direct implications for the design of workplace mental health programs. Understandings of how digital CBT-I might be optimized were advanced by considering not only intervention content but also the context in which it is delivered. Thermoregulatory physiology was brought into direct clinical conversation with sleep disorders, identifying a dimension of treatment that remains largely untapped in routine practice. The scarcity of evidence for non-pharmacological treatment in narcolepsy was documented, and bruxism was positioned within a broader sleep-health framework, reinforcing the role of shared arousal and emotional dysregulation mechanisms. Finally, the reach of circadian medicine was extended to include eating disorder vulnerability, demonstrating that chronotype has psychiatric consequences that extend well beyond mood and sleep.

Several cross-cutting methodological lessons also emerge. The predominance of cross-sectional and self-report designs limits causal inference throughout, objective sleep measurement—actigraphy or polysomnography—was largely absent, and sample diversity remains a challenge: white, female higher-education participants are overrepresented in most studies. The time is right for the field to invest in longitudinal, multi-site, adequately powered trials that include racial, ethnic, and occupational diversity, validated objective sleep measures, and patient-reported outcomes that capture real-world functional impact.

Looking ahead, several research priorities stand out. First, the clinical management of Long COVID insomnia requires randomized trial evidence. CBT-I has demonstrated efficacy across a range of comorbid populations, including those with cardiovascular disease and multiple sclerosis [18,19,20,21], and pilot investigation in Long COVID is now warranted. Second, the interplay between circadian misalignment, immune dysregulation, and mental health deserves mechanistic attention: the hypothalamic–pituitary–adrenal axis dysfunction proposed as a neurobiological substrate of Long COVID insomnia [10] shares features with the circadian disruption pathways implicated in mood disorders [8], suggesting a common vulnerability architecture that might be targeted pharmacologically or behaviorally. Third, scalable, accessible delivery models for insomnia interventions must be developed and rigorously tested, especially for populations with structural barriers to care. The embedded dCBT-I model piloted by Lenoir et al. [13] offers one promising direction; smartphone-based approaches represent another [22]. Fourth, occupational sleep medicine [23,24,25] must move from description to intervention. The psychosocial mediators identified in nursing samples—anger, cynicism, nightmare distress—are amenable to targeted interventions such as imagery rehearsal therapy for nightmares, anger management training, and organizational-level changes to reduce moral injury and interpersonal injustice [26,27]. Randomized trials testing bundled interventions against insomnia as the primary outcome would fill a meaningful gap [28,29]. Fifth, thermoregulatory profiling (CBT, DPG, melatonin onset) should be incorporated into clinical phenotyping to guide personalized selection of chronotherapeutic and thermal interventions. Wearable sensor technology now makes ambulatory CBT monitoring feasible [14], and its integration into clinical trials would substantially advance this agenda. Sixth, literature on therapeutic interventions for narcolepsy requires significantly larger, properly controlled, long-term clinical trials, with a particular focus on the psychopathological burden of this condition, which includes depression and anxiety in approximately one-third of individuals suffering from the disease [15]. Eating disorder prevention programs should incorporate chronotype screening, and future intervention studies should test whether circadian alignment strategies (timed light exposure, melatonin, CBT-I) reduce ED risk in at-risk evening chronotypes [17].

The broader conceptual message of this Special Issue is perhaps its most important contribution: sleep and circadian rhythm disruption are not epiphenomena of disease but constitutive elements of its pathophysiology and course [30]. A theme that cuts across all contributions is the potential of sleep and circadian dysregulation as transdiagnostic processes—mechanisms that are not constrained by traditional diagnostic boundaries but instead operate across depressive disorders, anxiety, eating disorders, neurodevelopmental conditions, and chronic somatic illness. This transdiagnostic perspective is clinically consequential: it argues for routine sleep and circadian assessment as an integrative component of psychiatric and medical evaluation, rather than as a domain to be addressed only when sleep complaints are foregrounded by the patient. Across Long COVID, occupational health, narcolepsy, bruxism, thermoregulation, and eating disorders, the same thesis recurs—disorganized sleep undermines virtually every other system the clinician is trying to protect. Recognition of this fact should prompt greater integration of sleep medicine into mental health services, and encourage the development of transdiagnostic sleep interventions—particularly adaptations of CBT-I—designed to address circadian misalignment and sleep disturbance as shared risk factors [31]. The fields of consultation–liaison psychiatry and integrated medicine would do well to place sleep assessment and management at the front, rather than the margin, of clinical encounters.
